# Pineal region tumors

**DOI:** 10.3171/2021.4.FOCVID2174

**Published:** 2021-07-01

**Authors:** Frederick A. Boop, Jeffrey N. Bruce, Uğur Türe, David J. Daniels

**Affiliations:** 1 Department of Neurosurgery, University of Tennessee Health Science Center, Memphis;; 2 Semmes-Murphey Neurologic and Spine Institute, Memphis, Tennessee;; 3 Department of Neurosurgery, Columbia University, New York, New York;; 4 Department of Neurosurgery, Yeditepe University School of Medicine, Istanbul, Turkey; and; 5 Departments of Neurosurgery and Molecular Pharmacology, Mayo Clinic, Rochester, Minnesota

## INTRODUCTION

The neurosurgical management of lesions in the pineal region has always presented a challenge. From early days, the pineal gland was referred to as “the seat of the soul” by philosopher René Descartes. This was due to its location deep within the brain and the fact that it was single and mobile. Embryologically, the pineal region is formed by anlage from all three germ cell layers, making pathologies of the region disparate. Early attempts at surgery by Walter Dandy and contemporaries for pineal pathologies were fraught with unacceptable mortality because of their deep location and delicate surrounding anatomy. It was not until 1939, when Arne Torkildsen reported shunting the acquired hydrocephalus and giving radiation to pineal tumors, that survival began to improve. The introduction of improved magnification and illumination by M. Gazi Yaşargil, Bennett Stein, Albert Rhoton, and others led to rapid improvement in survival for pineal surgery such that, in the modern microsurgical era, patient death from pineal region surgery has become a sentinel event. Over the ensuing years, microsurgical techniques to remove these tumors have been expanded and refined. In this *Neurosurgical Focus: Video* issue, master neurosurgeons from around the world have contributed microsurgical videos of current surgical approaches to pineal pathologies that we hope you will find informative. May your patients be better because of them!

